# CT volume of enhancement of disease (VED) can predict the early response to treatment and overall survival in patients with advanced HCC treated with sorafenib

**DOI:** 10.1007/s00330-020-07171-3

**Published:** 2020-08-22

**Authors:** S. Colagrande, L. Calistri, C. Campani, G. Dragoni, C. Lorini, C. Nardi, A. Castellani, F. Marra

**Affiliations:** 1 Department of Experimental and Clinical Biomedical Sciences, Radiodiagnostic Unit n. 2, University of Florence - Azienda Ospedaliero-Universitaria Careggi, Largo Brambilla 3, 50134 Florence, Italy; 2 Department of Experimental and Clinical Medicine, University of Florence, 50134 Florence, Italy; 3 Department of Health Science, University of Florence, Viale Morgagni 48, 50134 Florence, Italy; 4 Research Centre Denothe, University of Florence, Florence, Italy

**Keywords:** Hepatocellular carcinoma, Sorafenib, Computer-assisted image analysis, Therapy, Treatment efficacy

## Abstract

**Objectives:**

To analyse the predictive value of the volume of enhancement of disease (VED), based on the CT arterial enhancement coefficient (ΔArt%), in the evaluation of the sorafenib response in patients with advanced hepatocellular carcinoma (HCC).

**Methods:**

Patients with sorafenib-treated advanced HCC, who underwent a multiphase contrast-enhanced CT before (T0) and after 60–70 days of starting therapy (T1), were included. The same target lesions utilised for the response evaluation according to modified Response Evaluation Criteria in Solid Tumors criteria were retrospectively used for the ΔArt% calculation ([(HU_arterial phase_ − HU_unenhanced phase_) / HU_unenhanced phase_] × 100). ΔArt% was weighted for the lesion volume to obtain the VED. We compared VED_T0_ and VED_T1_ values in patients with clinical benefit (CB) or progressive disease (PD). The impact of VED, ancillary imaging findings, and blood chemistries on survival probability was evaluated.

**Results:**

Thirty-two patients (25 men, mean age 65.8 years) analysed between 2012 and 2016 were selected. At T1, 8 patients had CB and 24 had PD. VED_T0_ was > 70% in 8/8 CB patients compared with 12/24 PD patients (*p* = 0.011). Patients with VED_T0_ > 70% showed a significantly higher median survival than those with lower VED_T0_ (451.5 days vs. 209.5 days, *p* = 0.032). Patients with VED_T0_ > 70% and alpha-fetoprotein_T0_ ≤ 400 ng/ml had significantly longer survival than all other three combinations. In multivariate analysis, VED_T0_ > 70% emerged as the only factor independently associated with survival (*p* = 0.037).

**Conclusion:**

In patients with advanced HCC treated with sorafenib, VED is a novel radiologic parameter obtained by contrast-enhanced CT, which could be helpful in selecting patients who are more likely to respond to sorafenib, and with a longer survival.

**Key Points:**

*• To achieve the best results of treatment with sorafenib in advanced HCC, a strict selection of patients is needed.*

*• New radiologic parameters predictive of the response to sorafenib would be essential.*

*• Volume of enhancement of disease (VED) is a novel radiologic parameter obtained by contrast-enhanced CT, which could be helpful in selecting patients who are more likely to respond to therapy, and with a longer survival.*

## Introduction

Hepatocellular carcinoma (HCC) is the fourth cause of cancer death in the world, with an increasing incidence, particularly in Western countries [1]. Many patients present with advanced stage disease, especially if the diagnosis is made outside of a surveillance program [2–4]. Sorafenib is a multi-kinase inhibitor, which interferes with neo-angiogenesis [2]. Its use, in the Sorafenib HCC Assessment Randomized Protocol (SHARP) and Asia Pacific trials [5, 6], induced a modest but significant increase in survival (3 months) with respect to the control group, although no radiologic evidence of response to therapy was reported [7]. Relevant side effects limit the use of this drug [5–8], and it would be crucial to identify specific biomarkers for therapy response prediction, currently not available, although several studies looked for computed tomography (CT) or magnetic resonance (MR) parameters to anticipate the response to treatment [9–11]. The arterial enhancement coefficient (ΔArt%) is a simple parameter, which provides information on the grade of tissue vascularisation by arterial phase evaluation of a standard contrast-enhanced CT. Choi et al [10] reported that changes in tumour vascularity were the most specific indicators of treatment response in patients with gastrointestinal stromal tumour on imatinib. Smith et al [12] made similar remarks for metastatic renal cell carcinomas on sorafenib or sunitinib. However, there are only few reports in the context of HCC [13].

In this study, we retrospectively evaluated the possible predictive value of the volume of enhancement of disease (VED), a new radiologic parameter based on ΔArt%, in predicting early response to treatment and survival in a group of patients with advanced HCC treated with sorafenib.

## Materials and methods

### Patients

The ethics committee of our institution approved this retrospective study on 27 January (ref 2016-435; OSS. 16-260). Each patient was assigned a numerical code to ensure the anonymity of the clinical data. Written informed consent was obtained for sorafenib treatment and for CT scans with contrast agent (CA) administration, according to the principles of the Declaration of Helsinki (revision of Edinburgh, 2000). Patients with advanced HCC followed in the hepatology division of our hospital and treated with sorafenib between October 2012 and May 2016 were considered. They were diagnosed with advanced HCC (BCLC-C) according to the European guidelines [2]. Patients underwent treatment with sorafenib at a dose of 400 to 800 mg/day. Only patients who had undergone contrast-enhanced CT examination before therapy (T0) and after 60–80 days of starting treatment (T1) at the local institution were considered. Patients with less than 45 days of treatment or patients with *target* lesion not evaluable (e.g. nodules < 1 cm) were excluded from the study.

### CT acquisition

All CT exams were performed with a standard protocol, using a 64-row detector scanner (Somatom Sensation CT, Siemens Medical Systems). The images were obtained in the cranial–caudal direction with breath-hold helical acquisition. The scanning parameters were 1.2 × 24 collimation, 120 kV (peak), 140–240 mAs (using automated dose modulation), 5.0 mm slice thickness with a reconstruction interval of 2.0 mm, pitch 1.2 and 0.5 s gantry rotation time. All patients received intravenous non-ionic CA (Ultravist 370, Bayer HealthCare Pharmaceuticals; 370 mg of iodine/1 ml), at a volume of 1.4 ml/kg of body weight, by a bolus at 3 ml/s, using a mechanical power injector (Medrad Stellant CT Injection System), followed by a 40 ml saline flush through a 20-G catheter inserted into an antecubital vein. After unenhanced CT, the time-to-peak aortic enhancement was evaluated by an automatic bolus tracking technique (CARE Bolus CT, Siemens Medical Systems) to determine the optimal scanning delay for the arterial phase. The single-level monitoring low-dose scanning (20 mAs) was initiated 5 s after CA injection, and arterial phase scanning was started automatically 15 s after the trigger threshold (increase of 120 Hounsfield units (HU)) had been reached at the level of suprarenal abdominal aorta. Portal venous (extended to the chest and lower abdomen) and equilibrium-phase acquisitions were obtained at 70 s and 180 s, respectively.

### Evaluation of the response to sorafenib

The anonymised images were evaluated in consensus by two abdominal radiologists (10-year experienced), and if discordant, a consensus was reached through a joint review with the study coordinator (30-year experienced). Following the modified Response Evaluation Criteria in Solid Tumors (mRECIST) criteria, the patient’s baseline level was established, annotating the characteristic of eligible lesions as “target” and “non-target” [14]. Selection criteria of the target lesion(s) were diameter > 1 cm, easily measurable and well-defined margins, with intratumoural arterial enhancement; HCC lesions previously treated with locoregional treatments were selected if the lesion showed a well-delineated area of viable tumour (at least 1 cm in longest diameter) on the arterial phase; in the presence of multiple HCC nodules, a maximum of two target lesions was selected; all other lesions or sites of disease were considered non-target lesions, including malignant portal vein thrombosis and lymph nodes detected at the porta hepatis with short axis > 20 mm. At T1, overall patient response was a result of the combined assessment of target lesions, non-target lesions and new lesions [14]. We considered *new intrahepatic lesion*, the nodules ≥ 1 cm with arterial enhancement with or without washout. The appearance of one or more new lesions indicated progressive disease (PD) regardless of the result of the comparison of target and non-target lesions. For the purpose of this study, only two groups were considered: PD and clinical benefit (CB), the latter comprising complete response (CR), partial response (PR) and stable disease (SD) [14].

### VED calculation

**Fig. 1:**
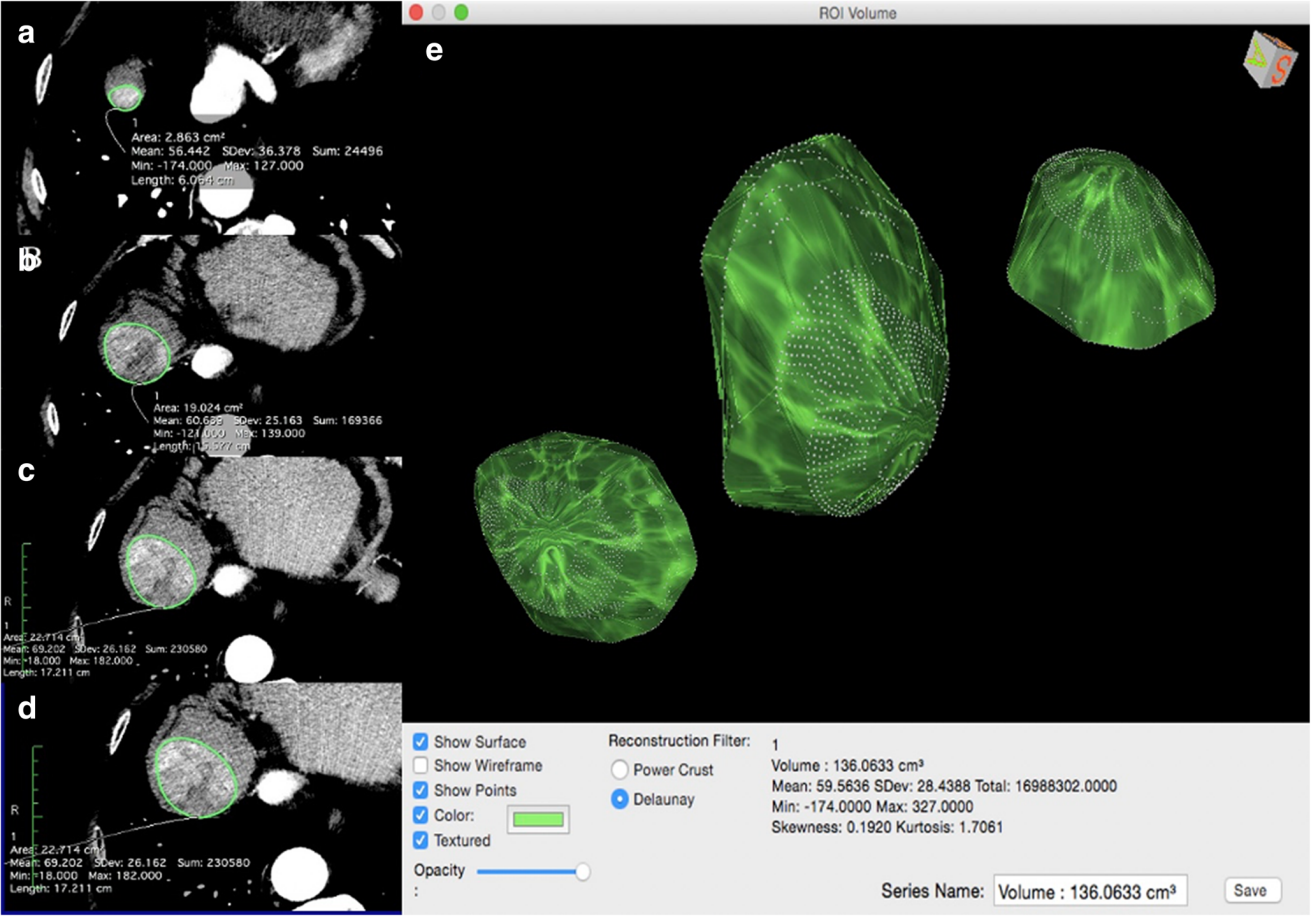
VED calculation. **a**–**d** In the 2D viewer, the ROI is marked on several arterial phase images with the “closed polygon” ROI tool (from the most caudal to the most cranial part of the lesion). Selecting the “ROI/ROI volume/Generate missing ROIs,” ROIs from the slices not included in the previous selection were generated. **e** After adjusting the contours of the lesion, if necessary, the “ROI/ROI volume/Compute volume” tool is used to obtain the 3D reconstruction, the volume and the mean density in the arterial phase (HU arterial phase) of the selected lesion, by summing the areas of all the ROIs, both selected and generated. After this, the operator copies the ROI of each arterial phase image and pastes it on the same-level unenhanced image. So, the estimation of the mean density at unenhanced phase (HU unenhanced phase) is obtained (not shown in the figure)

After the definition of the response to therapy, in a second session 15 days apart, the readers reviewed the images to calculate the volume of the liver target lesions, their arterial enhancement rate and the VED. If the reviewers were disagreeing, they reached a consensus through a joint review of the recorded images together with the coordinator. The same liver target lesions utilised for the assessment of response according to mRECIST criteria were used for the VED calculation. If more than two lesions were present, the largest were chosen to evaluate a quantity of disease in any case greater than 80%. The volume of the entire lesion, including necrotic areas, was calculated using OsiriX, an open-source Digital Imaging and Communications in Medicine (DICOM) viewer (Fig. 1). The degree of arterial enhancement was assessed at T0 and T1 time points according to the following formula:

$$\Delta \mathrm{Art}\%=\left[\left({\mathrm{HU}}_{\mathrm{arterial}\ \mathrm{phase}}-{\mathrm{HU}}_{\mathrm{unenhanced}\ \mathrm{phase}}\right)/{\mathrm{HU}}_{\mathrm{unenhanced}\ \mathrm{phase}}\right]\times 100$$

Therefore, to weight the ΔArt% of each lesion for the volume of the lesion itself, the new parameter, i.e. the VED, was calculated as follows: volume lesion × ΔArt% / volume lesion. While for a single target lesion ΔArt% = VED, when two target lesions were present, to take into account the possibility of heterogeneous behaviour of them, the VED was calculated according to the following formula:

$$\left(\mathrm{V}1\times \Delta \mathrm{Art}\%1\times \mathrm{V}2\times \Delta \mathrm{Art}\%2\right)/\left(\mathrm{V}1+\mathrm{V}2\right)\times 100$$

where V1 is volume lesion 1, ΔArt%1 is enhancement coefficient lesion 1, V2 is volume lesion 2 and ΔArt%2 is enhancement coefficient lesion 2.

The VED was calculated for each patient, both at baseline (VED_T0_) and after therapy (VED_T1_).

To evaluate the possible changes in mean enhancement of disease during sorafenib treatment, we calculated the ΔVED, applying the following formula: ΔVED = VED_T1_ − VED_T0_. The patients were classified as ΔVED_(pos)_, if VED_T1_ > VED_T0_, and as ΔVED_(neg)_, if VEDT_1_ < VED_T0_. Finally, we compared the volume and VED values between CB and PD patients at T0 and T1 time points. To avoid the possible reproducibility bias due to CA administration rate variation, HU arterial/unenhanced phase values of cancer-free parenchyma have been calculated from an average of 3 circular ROIs (1 cm in diameter) inserted on 3 consecutive slices on the parenchyma surrounding the lesion. So, the ΔArt% of the parenchyma was evaluated at T0 and T1 time points, for each patient.

### Ancillary imaging findings and blood chemistries

The presence of malignant portal vein thrombosis, distant metastases and the diameter of enlarged lymph nodes were assessed in all patients. Lymph nodes located at the level of the hepatic hilum were considered as metastatic in the case of a minor axis > 2 cm [14]. The values of the alpha-fetoprotein (AFP) and other serum parameters (total bilirubin, alkaline phosphatase, platelets, gamma glutamyl transferase, alanine aminotransferase, aspartate aminotransferase, international normalised ratio) were evaluated prior to and after therapy for CB and PD patients.

### Prediction of the therapy outcome and patient survival time

Survival time was evaluated for the study population and for each patient group. We tried to detect a VED_T0_ *cutoff* value that allowed us to classify the patients in CB and PD groups, with significantly different survival days. Finally, we evaluated the VED_T0_ cutoff values, ancillary imaging findings and laboratory parameters that could influence survival.

### Statistical analysis

Data were analysed using the SPSS® v.24.0 statistical analysis software (IBM Corp., 131; formerly SPSS Inc.) and Stata/IC 11 (StataCorp). For each variable, normality was evaluated using the Kolmogorov–Smirnov test. Since all the variables were not normally distributed, non-parametric tests (Mann–Whitney *U* test and Kruskal–Wallis statistical test for independent samples, Wilcoxon signed-rank test for correlated sample, McNemar’s test for paired proportions) were used to compare the distributions between subgroups or between subjects at T0 and T1 time points. Receiver operating characteristic (ROC) curves were used to find the best cutoff value of VED_T0_ to discriminate CB from PD patients. The area under the ROC curve was used as predictive power of the test. For different VED_T0_ cutoff values (from 10 to 110, in steps of 10), we evaluated patients’ survival, at T0 and T1 time points. Kaplan–Meier curves were used to graphically depict survival probabilities. Survival in different groups (VED_T0_ > 70; VED_T0_ ≤ 70) was compared, using the log-rank test. Moreover, given that AFP serum levels > 400 ng/ml are considered as diagnostic and specific for HCC [15], we compared the survival with the log-rank test in the following patient groups: AFP > 400 and VED_T0_ > 70, AFP ≤ 400 and VED_T0_ > 70, AFP > 400 and VED_T0_ ≤ 70, and AFP ≤ 400 and VED_T0_ ≤ 70. Univariate and multivariate linear regression analyses were used to evaluate the predictive value of VED and AFP (independent variables) for survival (outcome variable). Specifically, both independent variables were dichotomous for VED (> 70 or ≤ 70) and for AFP (> 400 or ≤ 400). For each analysis, a *p* value ≤ 0.05 was considered statistically significant.

## Results

### Patients’ characteristics and evaluation of the response to treatment

**Fig. 2:**
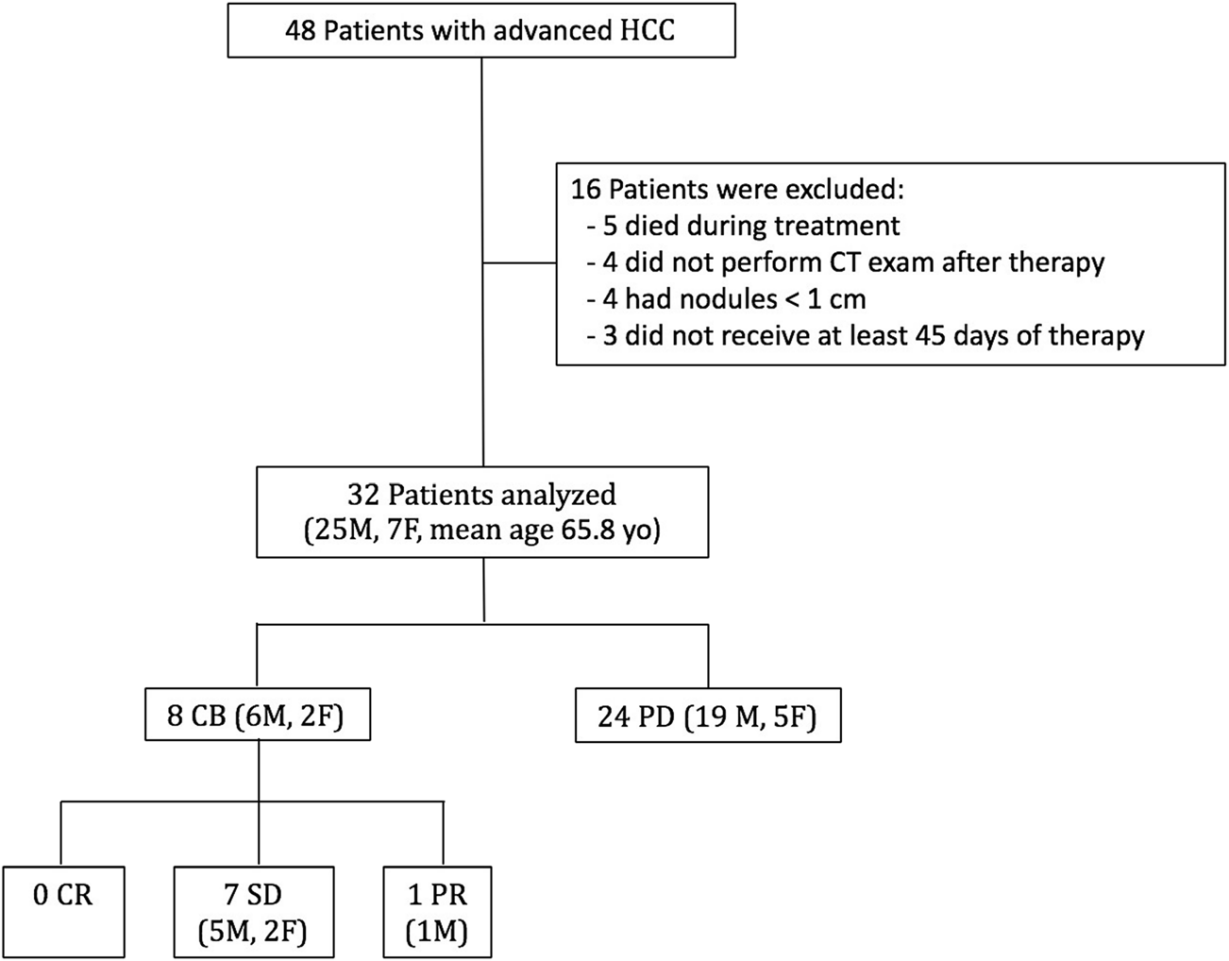
Patient disposition. HCC, hepatocellular carcinoma; CT, computed tomography; CB, clinical benefit; PD, progressive disease; CR, complete response; SD, stable disease; PR, partial response

**Fig. 3:**
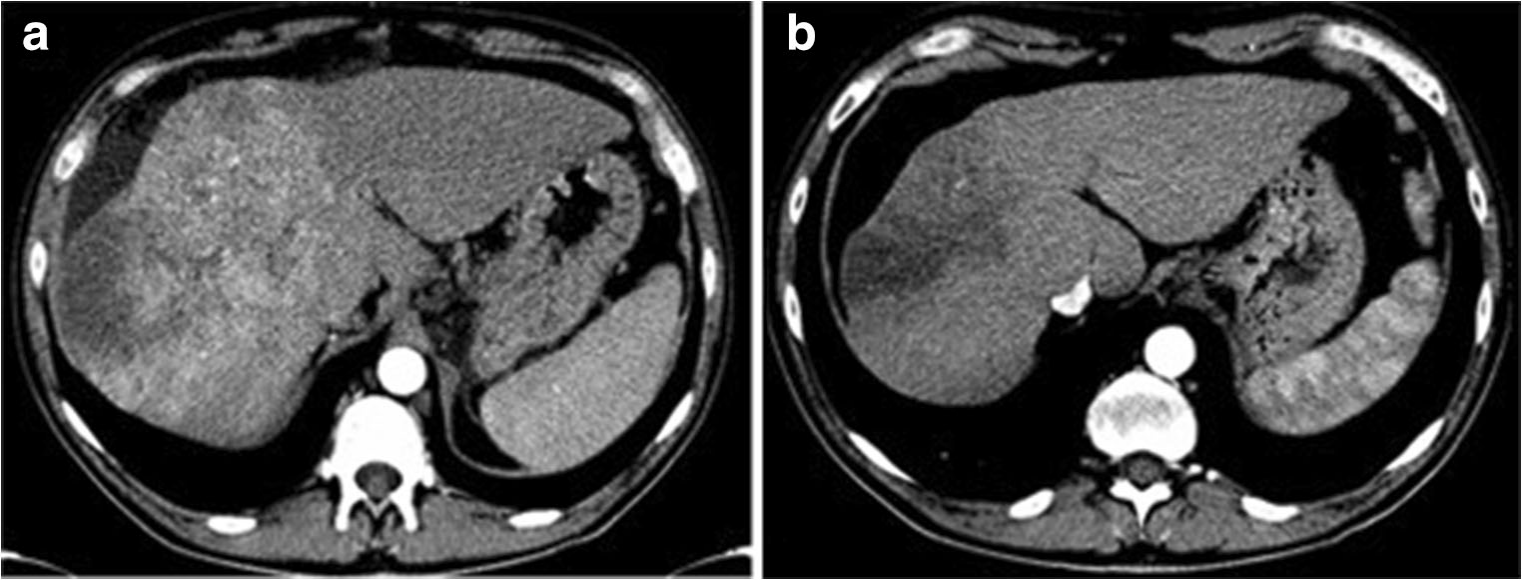
Representative images from a 51-year-old man with advanced HCC and partial response to sorafenib. **a** Arterial phase spiral CT scan at T0 time shows multiple merging nodular lesions in the right hepatic lobe with a marked enhancement in the arterial phase. **b** CT scan during arterial phase at T1 time shows a reduction in the size of the right hepatic lobe lesions, with significant lessening of vascularisation

Forty-eight patients with advanced HCC treated with sorafenib were initially assessed for eligibility (Fig. 2), and a total of 32 patients were included (11 patients with 1 nodule and 21 patients with 2 or more nodules). Characteristics of the 32 patients enrolled in the study are shown in Table 1. A maximum of 2 nodules was selected as target lesion for each patient, for a total of 53 nodules in 32 patients. The median duration of sorafenib therapy was 117 days (range, 45 to 255). The main adverse events during treatment were fatigue (16 patients), diarrhoea (13), hand–foot syndrome (13) and major worsening of liver function (3). At T1 control, the expert reader advice was required for 3 patients. No patients showed a CR, and 1 patient had a PR to therapy, with clear reduction in both size and enhancement (Fig. 3). The CB group included the latter, and 7 patients with SD. Twenty-four patients were in the PD group: ten PD patients showed a greater than 20% increase in the sum of the diameters of viable target lesions, one with 1 target lesion and 9 with 2 target lesions. In the other 14 patients, the sum of these diameters did not reach the 20% threshold as requested by mRECIST criteria to define a PD. However, in this subgroup, disease progression was due to appearance of new lesions and/or appearance or progression of neoplastic portal vein thrombosis (5 patients), lymph node involvement (5 patients) and distant metastases (4 patients).

**Table 1:** Characteristics of the 32 patients enrolled in the study

	Median (IQR) or *n* (%)
Age (years)	65.8 (63–78)
Gender (male)	25 (78.1)
Aetiology of chronic liver disease	
HCV	12 (37.5)
HBV	6 (18.8)
Alcohol	6 (18.8)
HBV-HDV	2 (6.2)
Cryptogenic	2 (6.2)
Primary biliary cholangitis	1 (3.1)
Non alcoholic steatohepatitis	3 (9.4)
Child–Pugh Score	
A5	11 (34.4)
A6	21 (65.6)
MELD Score	9 (7–11)
Extrahepatic spread (present)	4 (12.5)
Lymph node involvement	11 (34.4)
Portal vein thrombosis	10 (31.3)
Duration of therapy (days)	180 (90–270)

### Tumour volume and VED

**Fig. 4:**
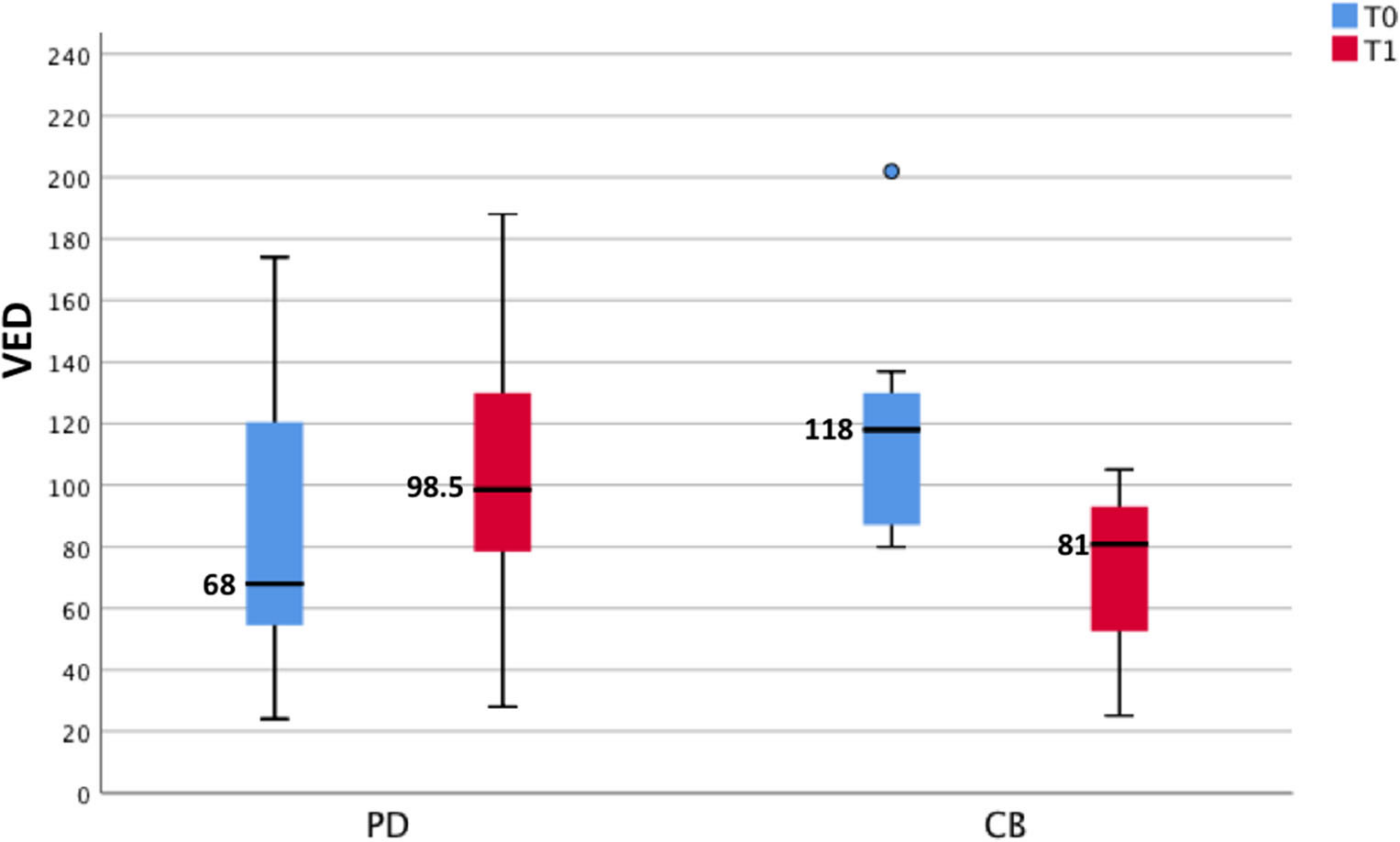
Box and whisker plot showing the distribution of VED (%) at T0 and T1 time points in CB and PD patients. Wilcoxon signed-rank test for the paired samples (T0 vs. T1 values) for PD patients (*p* = 0.097) and for CB patients (*p* = 0.018). Mann–Whitney *U* test for the independent samples (CB vs. PD) at T0 (*p* = 0.070) and at T1 (*p* = 0.064)

In CB patients, we found a reduction of about 15% in mean volume, while in PD patients, a significant increase in the volume of the target lesions was found, with an average increase of about 84% (Table 2). Patients with CB had higher baseline VED values than those with PD (Fig. 4) although the significance level of this difference was only borderline, due to the high variability. However, in CB patients, the VED values at the T1 time point were significantly lower than those at T0 (*p* = 0.018) (Fig. 4). When the ΔVED parameter was analysed, all CB patients fell into the ΔVED_(neg)_ class, while this behaviour was observed in only 9 out of 24 (38%) patients with PD. ΔArt% values of cancer-free parenchyma for the PD and CB groups patients did not show statistically significant differences comparing the two time points.

**Table 2:** Volume of the target lesions at T0 and T1 time points, and survival time of the patients

	T0^a, b^	T1^a^	p*
Volume (cm^3^)			
All (*N* = 53)			
Mean ± SD	61.6 ± 24.4	96.7 ± 28.7	< 0.001
Median	28.7	44.5	
Range	0.9–1912.3	0.5–2572.4	
CB (*N* = 14)			
Mean ± SD	65.3 ± 11.5	54.2 ± 9.1	0.331
Median	47.6	44.5	
Range	8.1–1150.3	0.5–522.5	
PD (*N* = 39)			
Mean ± SD	61.4 ± 28.7	112.8 ± 54.2	< 0.001
Median	22.4	54.2	
Range	0.9–1912.3	0.9–2572.4	
Survival time (days)			
All (*N* = 32)			
Mean ± SD	416.3 ± 279.0		
Median	325.5		
Range	116–1166		
CB (*N* = 8)			
Mean ± SD	687.2 ± 351.3		
Median	817.5		
Range	180–1166		
PD (*N* = 24)			
Mean ± SD	326.0 ± 182.5		
Median	289.5		
Range	116–792		

*CB* clinical benefit, *PD* progressive disease

^a^For volumes: Mann–Whitney *U* test for the independent samples (CB vs. PD): *p* = 0.391 and *p* = 0.558

^b^For survival time: Mann–Whitney *U* test for the independent samples (CB vs. PD): *p* < 0.001

*Wilcoxon signed-rank test for the paired samples (T0 vs. T1 values)

### Ancillary imaging findings and blood parameters

At the T0 time point, 10 patients had portal vein thrombosis, 11 had lymph node involvement and 4 had metastases (3 with lung involvement and 1 with bone involvement). Comparing the presence/absence of the findings, no statistically significant differences were found between the 2 time points (Table 3). Blood parameters and their temporal trends are summarised (Table 4). Only the median values of aspartate aminotransferase were significantly different comparing the pre/post-therapy values. In PD patients, both aminotransferase and bilirubin values were significantly different.

**Table 3:** Presence of portal vein thrombosis, lymph nodes and metastases, at T0 and T1 time points

Accessory imaging	T0, *N* (%)	T1, *N* (%)	*p* (McNemar’s test for paired proportions)
All patients			
Portal vein thrombosis	10 (31.3)	12 (37.5)	0.500
Lymph nodes	11 (34.4)	13 (40.6)	0.500
Metastases	4 (12.5)	6 (18.8)	0.500
CB patients			
Portal vein thrombosis	2 (25)	2 (25)	1.000
Lymph nodes	3 (37.5)	3 (37.5)	1.000
Metastases	0	0	1.000
PD patients			
Portal vein thrombosis	8 (33.3)	10 (41.7)	0.500
Lymph nodes	8 (33.3)	10 (41.7)	0.500
Metastases	4 (16.7)	6 (25)	0.500

*CB* clinical benefit, *PD* progressive disease

**Table 4:** Blood parameters at T0 and T1 time points

	T0			T1			p*
	Mean ± SD	Median	Range	Mean ± SD	Median	Range	
All							
AFP	26,127.1 ± 122,419.9	32.3	0.9–695,203	8539.0 ± 19,161.7	25.7	2–73,604.2	0.213
BLR	1.3 ± 0.7	1.29	0.37–2.92	1.8 ± 1.4	1.17	0.06–6.2	0.079
ALP	165.6 ± 87.0	132.5	64–432	190.3 ± 101.1	167.0	53–480	0.299
PLT	155,687.5 ± 115,830.6	112,000	51,000–587,000	153,843.7 ± 115,739.7	105,000	47,000–569,000	0.891
GGT	187.3 ± 147.4	132	40–648	205.4 ± 148.0	160.5	40–589	0.334
ALT	52.4 ± 23.4	52	10–103	71.8 ± 84.3	55	15–504	0.072
AST	78.6 ± 38.8	71.5	27–209	119.6 ± 115.3	77	2–544	0.042
INR	1.3 ± 0.4	1.17	0.9–3	1.3 ± 0.5	1.16	0.9–3.44	0.750
CB							
AFP	87,324.9 ± 245,622.6	17.6	0.9–695,203	2476.4 ± 5614.6	17.7	2–16,013	0.463
BLR	1.4 ± 0.7	1.4	0.6–2.7	1.1 ± 0.6	1.0	0.4–2.1	0.092
ALP	184.2 ± 105.7	151	98–432	162.7 ± 68.3	163.5	53–277	0.484
PLT	186,500 ± 104,793.7	176,000	59,000–337,000	138,500 ± 119,734.2	99,000	47,000–426,000	0.093
GGT	207.4 ± 143.4	141.5	81–502	199.5 ± 149.9	166	67–538	0.866
ALT	54.1 ± 22.3	55	30–103	63.2 ± 25.8	59.5	34–98	0.161
AST	75.6 ± 25.9	72	1.1–1.3	69.9 ± 20.7	70.5	38–111	0.362
INR	1.2 ± 0.1	1.2	1.1–1.3	1.2 ± 0.1	1.2	1–1.4	0.673
PD							
AFP	5727.8 ± 100,052.5	44.4	3.6–32,701	10,559.8 ± 21,638.9	40.5	3.8–73,604.2	0.230
BLR	1.2 ± 0.7	1.2	0.37–2.92	2.0 ± 1.5	1.4	0.06–6.17	0.018
ALP	159.3 ± 81.5	126	64–375	199.4 ± 109.5	167.5	60–480	0.107
PLT	145,416.7 ± 119,586.2	101,500	51,000–587,000	158,958.3 ± 116,544.6	114,000	50,000–569,000	0.254
GGT	180.6 ± 151.1	132	40–648	207.3 ± 150.6	160.5	40–589	0.273
ALT	51.8 ± 24.2	49	10–96	74.7 ± 96.6	55	15–504	0.244
AST	79.6 ± 42.6	71.6	27–209	136.1 ± 129	109	2–544	0.010
INR	1.3 ± 0.4	1.1	0.9–3	1.3 ± 0.5	1.1	0.9–3.44	0.395

*AFP* alpha-fetoprotein, *BLR* total bilirubin, *ALP* alkaline phosphatase, *PLT* platelets, *GGT* gamma-glutamyl transferase, *ALT* alanine aminotransferase, *AST* aspartate aminotransferase, *INR* normalised international ratio

*Wilcoxon signed-rank test for the correlated sample

### Prediction of the response to therapy and patient survival time

**Fig. 5:**
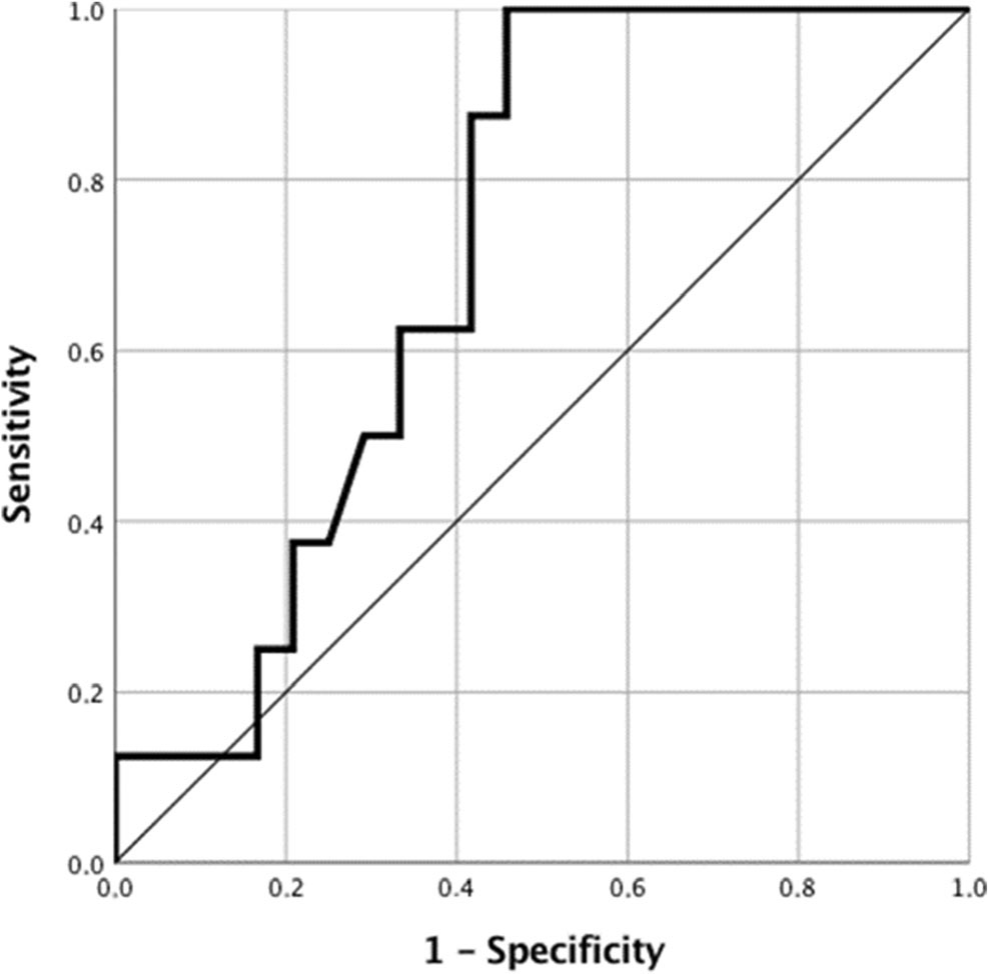
Receiver operating characteristic (ROC) curve. VED_T0_ value to discriminate CB from PD patients. The area under the ROC curve is 0.716

**Fig. 6:**
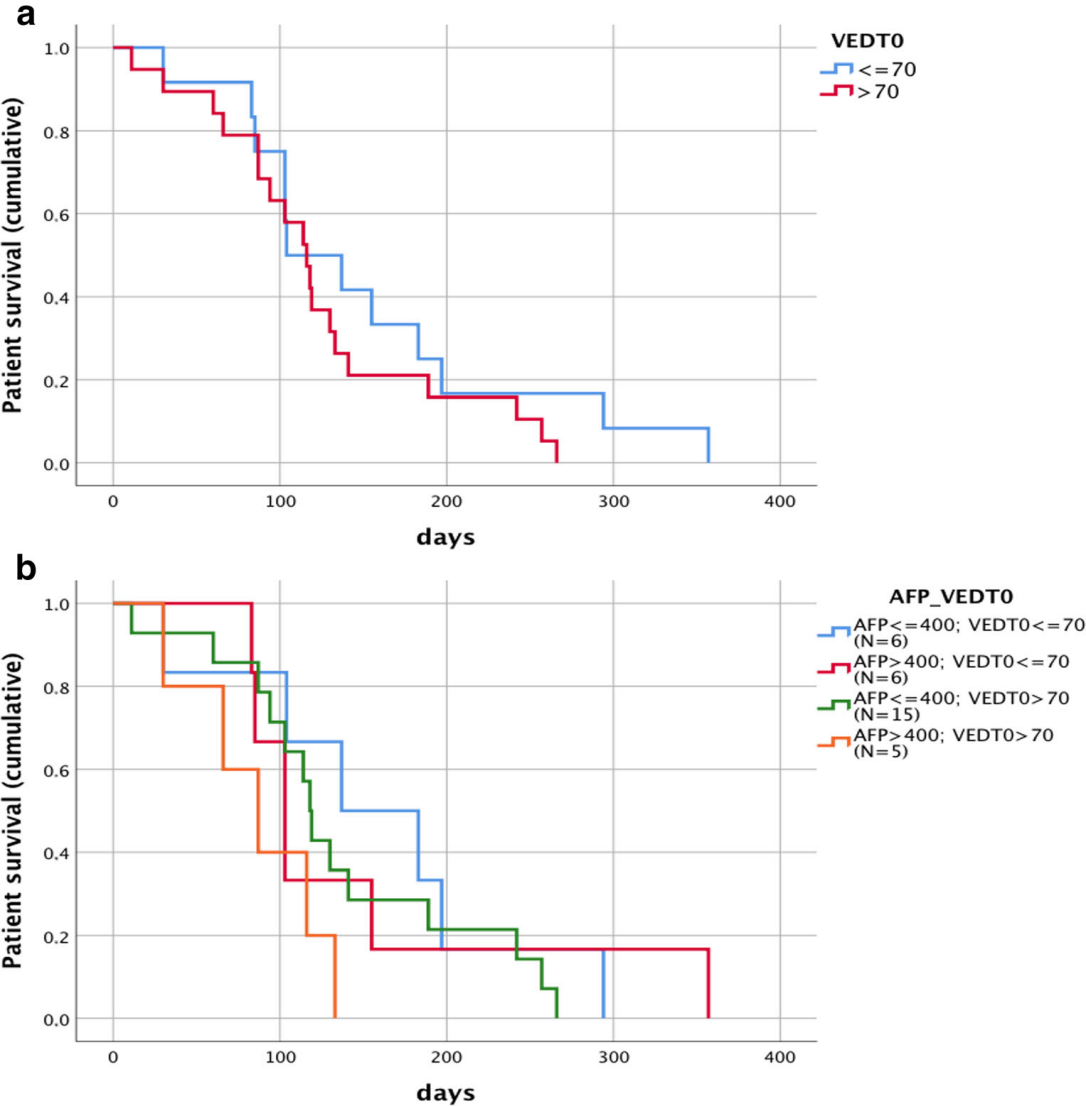
Survival analysis. Kaplan–Meier plots by the level of VED_T0_ (> 70, ≤ 70) (**a**) and by the level of AFT (> 400, ≤ 400) and VED_T0_ (> 70, ≤ 70) (**b**). For **a**, log rank (Mantel–Cox) = 0.007, Breslow (generalised Wilcoxon) = 0.024 and Tarone–Ware = 0.013. For **b**, log rank (Mantel–Cox) = 0.030, Breslow (generalised Wilcoxon) = 0.019 and Tarone–Ware = 0.024. In particular, survival time (mean) is as follows: if VED_T0_ > 70% and AFP_T0_ ≤ 400 ng/ml, 582 days, and AFP_T0_ > 400 ng/ml, 208 days; and if VED_T0_ ≤ 70% and AFP_T0_ ≤ 400 ng/ml, 213 days, and AFP_T0_ > 400 ng/ml, 213 days

To evaluate the significance of VED in the prediction of the outcome of therapy, ROC curves (Fig. 5) showed that a VED_T0_ cutoff value of 70% had the highest sensitivity and specificity (100% and 54.2%, respectively) in discriminating CB from PD patients. Survival time from the beginning of sorafenib therapy was highly variable (from 4 months to more than 2 years), and it was significantly longer in CB vs. PD patients (*p* = 0.001, Table 2). Separating the patients according to different cutoff values of VED_T0_, those with VED_T0_ > 70% showed a significantly longer survival time than those with lower VED_T0_ (506 ± 306 days vs. 266 ± 133 days, *p* = 0.032; Fig. 6, Table 5). Additionally, patients with ΔVED_(neg)_ showed a tendency to an average survival time longer than those with ΔVED_(pos)_ (493 ± 319 days vs. 328 ± 201 days, *p* = 0.189). At T0, the presence of portal vein thrombosis, lymph nodes or metastases did not significantly influence survival (*p* = 0.411, *p* = 0.327 and *p* = 0.564, respectively). Among blood parameters, only AFP at T0 significantly influenced survival time. Twenty-one patients with AFP_T0_ ≤ 400 ng/ml showed an average survival of 478 ± 282 days, while in 11 patients with AFP_T0_ > 400 ng/ml, survival was 299 ± 243 days (*p* = 0.02). When VED_T0_ values and AFP levels were combined, median survival was significantly longer in patients with VED_T0_ > 70% and AFP_T0_ ≤ 400 ng/ml than in all other combinations (Fig. 6). A multivariate linear regression analysis clarified the role and the weight of the baseline VED and AFP, in predicting survival. The results showed that VED_T0_ > 70% predicts a longer survival (*β* = 209.6; *p* = 0.037), while AFP lost its predictive role (*p* = 0.216).

**Table 5:** Survival time by different cutoff values of VED, at T0 and T1 time points

VED cutoff values	T0			T1		
	*N*	Survival time (mean ± SD)	p*	*N*	Survival time (mean ± SD)	p*
> 110	13	469.1 ± 342.1	0.791	10	293.3 ± 122.3	0.269
≤ 110	19	380.2 ± 229.5		22	472.3 ± 313.1	
> 100	14	463.9 ± 329.3	0.667	12	335.7 ± 208.8	0.366
≤ 100	18	379.3 ± 236.1		20	464.7 ± 308.5	
> 90	15	471.7 ± 318.8	0.478	17	345.8 ± 206.0	0.261
≤ 90	17	367.5 ± 237.8		15	496.3 ± 333.1	
> 80	18	485.3 ± 309.5	0.180	22	346.6 ± 218.7	0.060
≤ 80	14	327.7 ± 213.1		10	569.7 ± 344.3	
> 70	20	506.5 ± 306.2	0.032	24	387.8 ± 251.6	0.334
≤ 70	12	266.0 ± 133.9		8	502.0 ± 354.3	
> 60	22	491.5 ± 295.3	0.018	26	411.1 ± 288.2	0.588
≤ 60	10	250.9 ± 142.7		6	439.0 ± 257.8	
> 50	28	442.5 ± 287.1	0.169	26	411.1 ± 288.2	0.588
≤ 50	4	233.5 ± 107.9		6	439.0 ± 257.8	

*Mann–Whitney *U* test for the independent samples

## Discussion

After its approval in 2008, sorafenib remained the only first-line treatment for advanced HCC, until the recent approval of lenvatinib [16]. Clinical experience accumulated during these years [17–19] indicates that sorafenib improves overall survival of patients with HCC, in the absence of objective response, and that tumour progression is better used as a surrogate of survival. However, to achieve the best results with sorafenib treatment of advanced HCC, a strict selection of patients is needed. Therefore, considerable efforts have been made to identify baseline factors that could predict the response to sorafenib. Minor advances were made with a few biohumoral factors weakly associated with a good response to therapy, while no molecular markers of response were identified [20–22]. Patients undergoing sorafenib therapy are often elderly, and the therapy is associated with important side effects, yet affording a limited survival advantage over untreated patients.

In this study, we identified the VED, a parameter based on the degree of arterial enhancement of HCC nodules, weighed by the volume of the target lesion(s), as a relevant factor in the prediction of the response to sorafenib. Several data from our study support the potential utility of this new parameter in the management of patients with HCC. We observed that CB patients tended to have a higher mean VED at baseline and a significant decrease in VED was found at the T1 time in CB patients, as compared with PD, suggesting that a positive outcome of sorafenib therapy is associated with a reduction in this parameter. Importantly, all CB patients fell in the group with higher VED_T0_, i.e. > 70%. These data are strongly supported by the analysis of survival in the different groups. In fact, median survival in the VED_T0_ > 70% group was almost twice longer than that in patients with lower VED as baseline. In contrast, none of the patients with a VED_T0_ ≤ 70% had a CB from sorafenib therapy. Therefore, this parameter might be especially useful to identify the patients who are not likely to respond, characterised by low basal VED. Conversely, among patients with VED_T0_ > 70%, 12 out of 20 still did not respond to treatment.

Our results support the hypothesis that low-vascularised HCC nodules are poorly sensitive to sorafenib therapy. This assumption is biologically plausible based on the pharmacological properties of sorafenib, whose main mechanism of action is the reduction of neo-angiogenesis, inhibiting the activity of vascular endothelial and platelet-derived growth factors [23]. In the treatment of other neoplastic diseases, high expression of these factors and/or increased activity of their cognate receptors makes the drug more likely to be effective [24–27]. Thus, a high pre-therapy VED may be viewed as a proxy for a high pro-angiogenic activity targeted by sorafenib. In agreement, CB patients had a significant reduction in VED at T1.

Our study also provides additional evidence for the negative prognostic role of AFP elevation [28]. In fact, patients with AFP > 400 ng/ml had a significantly shorter survival than the others. Combining the VED and AFP values at T0 allowed us to stratify the patients, where those with VED_T0_ > 70% and AFP_T0_ < 400 ng/ml were more likely to respond, showing an average survival of 17 months (vs. less than 10 months of the other groups) (Fig. 6).

The VED parameter is non-invasive, economic, fast and easy to calculate in standard CT acquisitions, even without specific hardware and software, and it could provide a semi-quantitative and reliable evaluation of the volume of the disease and its perfusion. The reproducibility of VED computation is influenced by various factors, such as the concentration, amount and flow rate of CA administered, in association with the characteristics of each patient, mainly cardiac function. Therefore, it is important to underline that in our study, the dynamic CT was acquired with the *bolus tracking* technique to ensure that enhanced phases were as comparable as possible among patients. The lack of statistically significant variation of the ΔArt% value in the non-focal parenchyma at T0 and T1 time points supports the high reproducibility of this method.

The possible predictive role of MR diffusion and perfusion techniques in sorafenib-treated patients was evaluated [29–36]. Perfusion CT allows quantitative analysis of various parameters related to the micro-vascularisation of a neoplasm [37–39] and seems able to disclose significant differences between tumour tissue and surrounding parenchyma [30, 40]. However, perfusion CT is poorly reproducible and requires a software program which is not widely available, and the radiation dose administered is considerably higher than that of standard CT acquisitions. Also, dynamic contrast-enhanced ultrasound was investigated in evaluating the effectiveness of anti-angiogenic drugs on tumour perfusion in patients with HCC with encouraging results [41–45].

Some limitations of this study should be acknowledged. Firstly, the patients are few, and extension of these results to a larger series is warranted; however, each one was followed till his death. Also, the study has been conducted in a single centre, and reproducibility in other non-specialised facilities should be assessed. Then, to classify CB vs. PD patients, we could not use LI-RADS v2018 (not dealing with the evaluation of the response to systemic treatment). Thus, we chose mRECIST [14], even if we were conscious of some bias related to these criteria (non-volumetric evaluation, non-percentage assessment of the enhancement, incomplete evaluation reliability for systemic therapy) [8, 14, 16, 46–49], and that a revision of this system has been recently published by the same authors [50]. Furthermore, in this series, the VED was calculated for a maximum of 2 target lesions, which accounted for more than 80% of the overall disease burden. It should be pointed out that VED measurement may be difficult in some patients, especially when multifocal and infiltrative disease, with poor margins and different degrees of enhancement, is present. Finally, although all patients have been investigated with the same protocol and the same CT scanner, the retrospective nature of our work is an additional weakness.

In conclusion, this study identified the VED as a novel parameter obtained by a standard CT, which could be helpful, if confirmed by larger series in a prospective fashion, in predicting the response to treatment, identifying patients who are less likely to respond to sorafenib.

## Abbreviations

ΔArt%: Arterial enhancement coefficient
AFP: Alpha-fetoprotein
BCLC: Barcellona Clinic Liver Cancer
CA: Contrast agent
CB: Clinical benefit
CR: Complete response
HCC: Hepatocellular carcinoma
HU: Hounsfield units
mRECIST: Modified Response Evaluation Criteria in Solid Tumors
PD: Progressive disease
PR: Partial response
SD: Stable disease
SHARP: Sorafenib HCC Assessment Randomized Protocol
VED: Volume of enhancement of disease

## References

[1] Akinyemiju T, Abera S, Ahmed M (2017). The burden of primary liver cancer and underlying etiologies from 1990 to 2015 at the global, regional, and national level. JAMA Oncol.

[2] European Association for The Study of the Liver (2018). EASL clinical practice guidelines: management of hepatocellular carcinoma. J Hepatol.

[3] McGlynn KA, London WT (2012). International liver cancer incidence trends-letter. Cancer Epidemiol Biomarkers Prev.

[4] de Lope CR, Tremosini S, Forner A, Reig M, Bruix J (2012) Management of HCC. J Hepatol 56(Suppl 1):S75–S87 10.1016/S0168-8278(12)60009-9 22300468

[5] Llovet JM, Ricci S, Mazzaferro V (2008). Sorafenib in advanced hepatocellular carcinoma. N Engl J Med.

[6] Cheng AL, Kang YK, Chen Z (2009). Efficacy and safety of sorafenib in pts in the Asia-Pacific region with advanced hepatocellular carcinoma: a phase III randomised, double-blind, placebo-controlled trial. Lancet Oncol.

[7] Roberts LR (2008). Sorafenib in liver cancer: just the beginning?. N Engl J Med.

[8] Colagrande S, Regini F, Taliani GG, Nardi C, Inghilesi AL (2015) Advanced hepatocellular carcinoma and sorafenib: diagnosis, indications, clinical and radiological follow-up. World J Hepatol 7(8):1041–1053 10.4254/wjh.v7.i8.1041 PMC4450181 26052393

[9] De Schepper AM, Vanhoenacker F, Gielen J (2006). Imaging of soft tissue tumors.

[10] Choi H, Charnsangavej C, de Castro Faria S et al (2004) CT evaluation of the response of gastrointestinal stromal tumors after imatinib mesylate treatment: a quantitative analysis correlated with FDG PET findings. AJR Am J Roentgenol 183:1619–1628 10.2214/ajr.183.6.01831619 15547201

[11] Choi H (2005). Critical issues in response evaluation on computed tomography: lessons from the gastrointestinal stromal tumor model. Curr Oncol Rep.

[12] Smith AD, Lieber ML, Shah SN (2019) Assessing tumor response and detecting recurrence in metastatic renal cell carcinoma on targeted therapy: importance of size and attenuation on contrast-enhanced CT. AJR Am J Roentgenol 194:157–165 10.2214/AJR.09.2941 20028918

[13] Salvaggio G, Furlan A, Agnello F (2014). Hepatocellular carcinoma enhancement on contrast-enhanced CT and MR imaging: response assessment after treatment with sorafenib: preliminary results. Radiol Med.

[14] Lencioni R, Llovet JM (2010). Modified RECIST (mRECIST) assessment for hepatocellular carcinoma. Semin Liver Dis.

[15] de Lope CR, Tremosini S, Forner A, Reig M, Bruix J (2012) Management of HCC. J Hepatol 56(Suppl 1):S75–S87 10.1016/S0168-8278(12)60009-9 22300468

[16] Kudo M, Finn RS, Qin S (2018). Lenvatinib versus sorafenib in first-line treatment of patients with unresectable hepatocellular carcinoma: a randomised phase 3 non-inferiority trial. Lancet.

[17] Giannini EG, Bucci L, Garuti F (2018). Patients with advanced hepatocellular carcinoma need a personalized management: a lesson from clinical practice. Hepatology.

[18] Colagrande S, Inghilesi AL, Aburas S, Taliani GG, Nardi C, Marra F (2016) Challenges of advanced hepatocellular carcinoma. World J Gastroenterol 22:7645–7659 10.3748/wjg.v22.i34.7645 PMC5016365 27678348

[19] Marrero JA, Kudo M, Venook AP (2016). Observational registry of sorafenib use in clinical practice across Child-Pugh subgroups: the GIDEON study. J Hepatol.

[20] Llovet JM, Peña CE, Lathia CD (2012). SHARP Investigators Study Group: plasma biomarkers as predictors of outcome in patients with advanced hepatocellular carcinoma. Clin Cancer Res.

[21] Shao YY, Lin ZZ, Hsu C, Shen YC, Hsu CH, Cheng AL (2010) Early alpha-fetoprotein response predicts treatment efficacy of antiangiogenic systemic therapy in patients with advanced hepatocellular carcinoma. Cancer 116:4590–4596 10.1002/cncr.25257 20572033

[22] Hong YM, Yoon KT, Hwang TH, Heo J, Woo HY, Cho M (2019) Changes in the neutrophil-to-lymphocyte ratio predict the prognosis of patients with advanced hepatocellular carcinoma treated with sorafenib. Eur J Gastroenterol Hepatol 31:1250–1255 10.1097/MEG.0000000000001405 30925530

[23] Wilhelm SM, Carter C, Tang L (2004). BAY 43-9006 exhibits broad spectrum oral antitumor activity and targets the RAF/MEK/ERK pathway and receptor tyrosine kinases involved in tumor progression and angiogenesis. Cancer Res.

[24] Cao G, Li X, Qin C, Li J (2015) Prognostic value of VEGF in hepatocellular carcinoma patients treated with sorafenib: a meta-analysis. Med Sci Monit 21:3144–3151 10.12659/MSM.894617 PMC4617189 26476711

[25] Wilhelm SM, Adnane L, Newell P, Villanueva A, Llovet JM, Lynch M (2008) Preclinical overview of sorafenib, a multikinase inhibitor that targets both Raf and VEGF and PDGF receptor tyrosine kinase signaling. Mol Cancer Ther 7(10):3129–3140 10.1158/1535-7163.MCT-08-0013 PMC12261297 18852116

[26] Peña C, Lathia C, Shan M, Escudier B, Bukowski RM (2010) Biomarkers predicting outcome in patients with advanced renal cell carcinoma: results from sorafenib phase III treatment approaches in renal cancer global evaluation Trial. Clin Cancer Res 16(19):4853–4863 10.1158/1078-0432.CCR-09-3343 20651059

[27] Cho DC (2013). Prognostic biomarkers for patients with advanced renal cell carcinoma treated with VEGF-targeted tyrosine kinase inhibitors. Onco Targets Ther.

[28] Bruix J, Cheng AL, Meinhardt G, Nakajima K, De Sanctis Y, Llovet J (2017) Prognostic factors and predictors of sorafenib benefit in patients with hepatocellular carcinoma: analysis of two phase III studies. J Hepatol 67:999–1008 10.1016/j.jhep.2017.06.026 28687477

[29] Schraml C, Schwenzer NF, Martirosian P (2009) Diffusion-weighted MRI of advanced hepatocellular carcinoma during sorafenib treatment: initial results. AJR Am J Roentgenol 193(4):301–307 10.2214/AJR.08.2289 19770299

[30] Sacco R, Faggioni L, Bargellini I (2013). Assessment of response to sorafenib in advanced hepatocellular carcinoma using perfusion computed tomography: results of a pilot study. Dig Liver Dis.

[31] Mungai F, Pasquinelli F, Mazzoni LN (2014). Diffusion-weighted magnetic resonance imaging in the prediction and assessment of chemotherapy outcome in liver metastases. Radiol Med.

[32] Yeo DM, Choi JI, Lee YJ, Park MY, Chun HJ, Lee HG (2014) Comparison of RECIST, mRECIST, and Choi criteria for early response evaluation of hepatocellular carcinoma after transarterial chemoembolization using drug-eluting beads. J Comput Assist Tomogr 38:391–397 10.1097/RCT.0000000000000070 24681857

[33] Ronot M, Bouattour M, Wassermann J (2014). Alternative response criteria (Choi, European Association for the Study of the Liver, and Modified Response Evaluation Criteria in Solid Tumors [RECIST]) versus RECIST 1.1 in pts with advanced hepatocellular carcinoma treated with sorafenib. Oncologist.

[34] Pandharipande PV, Krinsky GA, Rusinek H, Lee VS (2005) Perfusion imaging of the liver: current challenges and future goals. Radiology 234:661–673 10.1148/radiol.2343031362 15734925

[35] Dai X, Schlemmer HP, Schmidt B (2013). Quantitative therapy response assessment by volumetric iodine-uptake measurement: initial experience in pts with advanced hepatocellular carcinoma treated with sorafenib. Eur J Radiol.

[36] Jiang T, Zhu AX, Sahani DV (2013). Established and novel imaging biomarkers for assessing response to therapy in hepatocellular carcinoma. J Hepatol.

[37] Sahani DV, Holalkere NS, Mueller PR, Zhu AX (2013) Advanced hepatocellular carcinoma: CT perfusion of liver and tumor tissue-initial experience. Radiology 243:736–743 10.1148/radiol.2433052020 17517931

[38] Hayano K, Lee SH, Yoshida H, Zhu AX, Sahani DV (2014) Fractal analysis of CT perfusion images for evaluation of antiangiogenic treatment and survival in hepatocellular carcinoma. Acad Radiol 21:654–660 10.1016/j.acra.2014.01.020 24703479

[39] Zhu AX, Holalkere NS, Muzikansky A, Horgan K, Sahani DV (2008) Early antiangiogenic activity of bevacizumab evaluated by computed tomography perfusion scan in pts with advanced hepatocellular carcinoma. Oncologist 13(2):120–125 10.1634/theoncologist.2007-0174 18305056

[40] Wang S, Zhou C, Zhao X (2010). Perfusion study of hepatic tumors: use of multi-detector row helical CT and liver perfusion software. World J Radiol.

[41] Zheng SG, Xu HX, Liu LN (2014). Management of hepatocellular carcinoma: the role of contrast-enhanced ultrasound. World J Radiol.

[42] Zocco MA, Garcovich M, Lupascu A (2013). Early prediction of response to sorafenib in pts with advanced hepatocellular carcinoma: the role of dynamic contrast enhanced ultrasound. J Hepatol.

[43] Shiozawa K, Watanabe M, Kikuchi Y, Kudo T, Maruyama K, Sumino Y (2012) Evaluation of sorafenib for hepatocellular carcinoma by contrast-enhanced ultrasonography: a pilot study. World J Gastroenterol 18:5753–5758 10.3748/wjg.v18.i40.5753 PMC3484345 23155317

[44] Mandai M, Koda M, Matono N (2011). Assessment of hepatocellular carcinoma by contrast-enhanced ultrasound with perfluorobutane microbubbles: comparison with dynamic CT. Br J Radiol.

[45] Sugimoto K, Moriyasu F, Saito K (2013). Hepatocellular carcinoma treated with sorafenib: early detection of treatment response and major adverse events by contrast-enhanced US. Liver Int.

[46] Kawaoka T, Aikata H, Murakami E (2012). Evaluation of the mRECIST and α-fetoprotein ratio for stratification of the prognosis of advanced-hepatocellular-carcinoma patients treated with sorafenib. Oncology.

[47] Horger M, Lauer UM, Schraml C (2009). Early MRI response monitoring of patients with advanced hepatocellular carcinoma under treatment with the multikinase inhibitor sorafenib. BMC Cancer.

[48] Tovoli F, Renzulli M, Negrini G (2018). Inter-operator variability and source of errors in tumour response assessment for hepatocellular carcinoma treated with sorafenib. Eur Radiol.

[49] Seo N, Kim MS, Park MS (2020). Evaluation of treatment response in hepatocellular carcinoma in the explanted liver with Liver Imaging Reporting and Data System version 2017. Eur Radiol.

[50] Llovet JM, Lencioni R (2020). mRECIST for HCC: performance and novel refinements. J Hepatol.

