# Correction to: CT volume of enhancement of disease (VED) can predict the early response to treatment and overall survival in patients with advanced HCC treated with sorafenib

**DOI:** 10.1007/s00330-020-07592-0

**Published:** 2020-12-15

**Authors:** S. Colagrande, L. Calistri, C. Campani, G. Dragoni, C. Lorini, C. Nardi, A. Castellani, F. Marra

**Affiliations:** 1 Department of Experimental and Clinical Biomedical Sciences, Radiodiagnostic Unit n. 2, University of Florence – Azienda Ospedaliero-Universitaria Careggi, Largo Brambilla 3, 50134 Florence, Italy; 2 Department of Experimental and Clinical Medicine, University of Florence, 50134 Florence, Italy; 3 Department of Health Science, University of Florence, Viale Morgagni 48, 50134 Florence, Italy; 4 Research Centre Denothe, University of Florence, Florence, Italy

**Correction to: European Radiology**

10.1007/s00330-020-07171-3

The original version of this article, published on 22 August 2020, unfortunately contained a mistake. The following correction has therefore been made in the original: The second equation in paragraph “VED calculation” was incorrect; the corrected equation is given below. The original article has been corrected.

$$ \left(\mathrm{V}1\times \Delta \mathrm{Art}\%1+\mathrm{V}2\times \Delta \mathrm{Art}\%2\right)/\left(\mathrm{V}1+\mathrm{V}2\right)\times 100 $$

